# Computer-Tailored Decision Support Tool for Lung Cancer Screening: Community-Based Pilot Randomized Controlled Trial

**DOI:** 10.2196/17050

**Published:** 2020-11-03

**Authors:** Lisa Carter-Harris, Robert Skipworth Comer, James E Slaven II, Patrick O Monahan, Emilee Vode, Nasser H Hanna, DuyKhanh Pham Ceppa, Susan M Rawl

**Affiliations:** 1 Memorial Sloan Kettering Cancer Center New York, NY United States; 2 Indiana University School of Informatics and Computing Indianapolis, IN United States; 3 School of Medicine Indiana University Indianapolis, IN United States; 4 Indiana University School of Nursing Indianapolis, IN United States

**Keywords:** lung cancer screening, informed decision-making, shared decision-making, patient decision aid, patient education

## Abstract

**Background:**

Lung cancer screening is a US Preventive Services Task Force Grade B recommendation that has been shown to decrease lung cancer-related mortality by approximately 20%. However, making the decision to screen, or not, for lung cancer is a complex decision because there are potential risks (eg, false positive results, overdiagnosis). Shared decision making was incorporated into the lung cancer screening guideline and, for the first time, is a requirement for reimbursement of a cancer screening test from Medicare. Awareness of lung cancer screening remains low in both the general and screening-eligible populations. When a screening-eligible person visits their clinician never having heard about lung cancer screening, engaging in shared decision making to arrive at an informed decision can be a challenge. Methods to effectively prepare patients for these clinical encounters and support both patients and clinicians to engage in these important discussions are needed.

**Objective:**

The aim of the study was to estimate the effects of a computer-tailored decision support tool that meets the certification criteria of the International Patient Decision Aid Standards that will prepare individuals and support shared decision making in lung cancer screening decisions.

**Methods:**

A pilot randomized controlled trial with a community-based sample of 60 screening-eligible participants who have never been screened for lung cancer was conducted. Approximately half of the participants (n=31) were randomized to view LungTalk—a web-based tailored computer program—while the other half (n=29) viewed generic information about lung cancer screening from the American Cancer Society. The outcomes that were compared included lung cancer and screening knowledge, lung cancer screening health beliefs (perceived risk, perceived benefits, perceived barriers, and self-efficacy), and perception of being prepared to engage in a discussion about lung cancer screening with their clinician.

**Results:**

Knowledge scores increased significantly for both groups with greater improvement noted in the group receiving LungTalk (2.33 vs 1.14 mean change). Perceived self-efficacy and perceived benefits improved in the theoretically expected directions.

**Conclusions:**

LungTalk goes beyond other decision tools by addressing lung health broadly, in the context of performing a low-dose computed tomography of the chest that has the potential to uncover other conditions of concern beyond lung cancer, to more comprehensively educate the individual, and extends the work of nontailored decision aids in the field by introducing tailoring algorithms and message framing based upon smoking status in order to determine what components of the intervention drive behavior change when an individual is informed and makes the decision whether to be screened or not to be screened for lung cancer.

**International Registered Report Identifier (IRRID):**

RR2-10.2196/resprot.8694

## Introduction

Lung cancer screening is recommended by the US Preventive Services Task Force (USPSTF) with a Grade B recommendation and offers the potential to detect lung cancer via low-dose computed tomography of the chest at an earlier stage when more treatment options exist [1]. However, lung cancer screening is complex because there are associated risks and potential harms that complicate the decision to screen [1]. False-positive results and incidental findings have the potential to lead to invasive testing that are possible in this type of cancer screening [1,2]. Because of these potential risks, lung cancer screening is a preference-sensitive decision and requires patient-clinician discussion and a shared decision-making process. Therefore, the USPSTF recommends, and Medicare requires, that the decision to screen for lung cancer be the result of a documented shared decision making and counseling visit between a patient and their clinician with the use of one or more decision aids [1,3].

Knowledge and awareness about lung cancer screening among the general population is extremely low [4,5]. In order to foster increased patient-clinician discussions about this relatively new screening option, it is essential to leverage new ways to both increase awareness and knowledge about lung cancer screening. In response to our prior work with screening-eligible individuals, our team developed a theoretically grounded, computer-tailored decision support tool titled LungTalk (1) to increase awareness about lung cancer screening and improve knowledge about lung health and lung cancer screening benefits and risks, and (2) prepare individuals to engage in discussions with their clinician in order to support the shared decision-making process.

In this paper, the results of a community-based, pilot randomized controlled trial to compare the effects of LungTalk to those of a nontailored lung screening information sheet in a sample of screening-eligible individuals are presented. LungTalk was developed using the USPSTF Lung Cancer Screening Guidelines as well as qualifying and certification criteria of the International Patient Decision Aid Standards instrument [6] as described in our protocol [7]. In addition to evaluating feasibility of the study procedures, the following research questions were answered:

1. Are there changes in knowledge of lung cancer risk and screening, and lung cancer screening health beliefs (perceived risk, perceived benefits, perceived barriers, self-efficacy) between patients who received LungTalk and those who received the nontailored lung screening information sheet?
2. Are there changes in participants’ perceptions of being prepared to engage in a discussion with their clinician about lung cancer screening between patients who received LungTalk and those who received the nontailored lung screening information sheet?
3. Are there changes in self-reported patient-clinician discussions about lung cancer screening and receipt of a lung cancer screening recommendation between patients who received LungTalk and those who received the nontailored lung screening information sheet?

## Methods

### Study Sample and Recruitment

Participants (n=60), both men and women, who were eligible for lung cancer screening were recruited using Facebook-targeted advertisement. (Facebook has the ability to “target” an advertisement by demographics and keywords listed in each individual Facebook user’s profile or interest list.) Using this technique, we were able to purposively sample people aged 55 years and older who indicated smoking as an interest or like in their profile. Frandsen et al [8] demonstrated that participants recruited to smoking cessation randomized clinical trials with Facebook advertisements did not differ from those recruited by traditional methods in either smoking characteristics or demographics. Our team extended Fransden’s [8] work by demonstrating that participants aged 55 years and older recruited to a web-based survey study for lung cancer screening did not differ from those recruited by traditional methods by either smoking characteristics or demographics thus demonstrating its utility to reach and recruit older long-term smokers [9]. A REDCap (Research Electronic Data Capture; Vanderbilt University) survey was used to screen and invite eligible participants to enroll in the study. Inclusion criteria mirrored the USPSTF Lung Cancer Screening Guidelines [1]: (1) aged 55 to 80 years; (2) 30 pack-year tobacco smoking history; (3) current smoker or former smoker who quit within the past 15 years; (4) not diagnosed with a condition that would be contraindicated for lung cancer screening; and (5) not diagnosed with lung cancer.

### Interventions

#### LungTalk Interactive Program

LungTalk is a computer-tailored decision support tool that is theoretically grounded in the Conceptual Model on Lung Cancer Screening Participation [10]. This model links the Health Belief Model [11] to the Precaution Adoption Process Model [12] and includes key psychological variables (eg, stigma, mistrust, fatalism, fear, and worry) as factors that may influence an individual’s decision to screen, or not, for lung cancer [10]. LungTalk is designed to increase knowledge and awareness about the option to screen, or not, for lung cancer and to prepare screening-eligible individuals to engage in shared decision making about lung cancer screening with their clinician. LungTalk educates high-risk individuals about (1) lung health broadly including the effects of nicotine; (2) risk factors for the development of lung cancer; (3) the option of lung cancer screening with low-dose computed tomography of the chest; and (4) the risks and benefits of lung cancer screening. Furthermore, because our prior research [4,13] revealed that messages needed to be different for needed to be different for individuals who currently versus used to smoke, messages in LungTalk are tailored by smoking status (see Multimedia Appendix 1).

LungTalk includes audio, video, and animation segments with tailoring algorithms for scripts presented from a master content library. In addition, LungTalk offers the option of saving or printing a tailored summary (at program completion) that individuals can use to guide a discussion with their clinician. This summary highlights key points related to lung health and screening tailored by smoking status, offers questions the user can ask to initiate a discussion with their clinician, and includes specific questions identified by the user that they wish to discuss with their clinician. Content in LungTalk is visually presented with text written at an eighth grade reading level. To meet the needs of people with low literacy and auditory preference learning styles, all content is narrated as well as shown as written text on screens (see Multimedia Appendix 1). Additional details on the development of LungTalk are described elsewhere [7].

#### Nontailored Lung Screening Information Sheet

The control group viewed a nontailored lung screening information sheet online that contained information compiled from lung cancer screening information developed by the American Cancer Society. The reading level of this written material was at an eighth grade level.

### Data Collection

Data collected between January 2017 and February 2017 from participants in the state of Indiana. Data were collected via online surveys completed by participants (baseline only) and telephone interviews conducted by trained research staff. The follow-up surveys were developed in REDCap, a secure web-based application to build and manage online surveys and databases. Participants completed a 20-minute baseline survey prior to randomization. Follow-up surveys were then completed 1 week and 3 months postintervention.

The baseline survey collected data on sociodemographic and health status characteristics, lung cancer and screening knowledge, lung cancer screening health beliefs (perceived risk of lung cancer, perceived benefits of, perceived barriers to, and self-efficacy for lung cancer screening) [11], perceived preparation to engage in a patient-clinician discussion about lung cancer screening, and stage of adoption for lung cancer screening. At completion of the baseline survey, each participant was randomized to receive either LungTalk or the nontailored lung screening information sheet. A link to either LungTalk or the lung screening information sheet was emailed to the participant based upon their randomization.

A follow-up telephone interview was conducted within 1 week of the participant completing the intervention. The interview included items to assess lung cancer and screening knowledge, lung cancer screening health beliefs (perceived risk, perceived benefits, perceived barriers, and self-efficacy) [11], satisfaction with the intervention, self-report of perception of preparation to engage in a patient-clinician discussion about lung cancer screening, and stage of adoption for lung cancer screening [12]. A second telephone interview was completed 3 months after receipt of the intervention to assess whether the participant had a subsequent discussion about lung cancer screening with a clinician, received a clinician recommendation for screening, and stage of adoption for lung cancer screening [12].

### Measures

Guided by the Conceptual Model on Lung Cancer Screening Participation, valid and reliable instruments were used to measure knowledge of lung cancer risk and screening, lung cancer screening health beliefs (perceived risk of lung cancer, perceived benefits of, perceived barriers to, and self-efficacy for lung cancer screening), health care clinician recommendation, perception of preparation to engage in a patient-clinician discussion about lung cancer screening, and stage of adoption for lung cancer screening participation. Stage of adoption was measured using an algorithm that is theoretically based upon the Precaution Adoption Process model [12]. The Precaution Adoption Process Model is supported by the Conceptual Model on Lung Cancer Screening Participation as an appropriate outcome variable in lung cancer screening decisions because it includes a stage to categorize individuals who have thoroughly weighed their options and decided not to be screened [12]. The Precaution Adoption Process Model categorizes individuals into 1 of 7 stages, including unaware, aware but unengaged, undecided, decided not to act, decided to act, action, and maintenance [14]. The Lung Cancer Screening Health Belief Scales (perceived risk, perceived benefits, perceived barriers, self-efficacy) had been previously validated in a community-based sample of 497 screening-eligible individuals [11]. *Total knowledge*, with Knowledge of Lung Cancer and Lung Cancer Screening, which is a 6-item multidimensional scale used in our preliminary study adapted from literature specific to lung cancer, will be assessed, including knowledge of lung cancer, risk, and screening; *total perceived risk*, with Perceived Risk of Lung Cancer Scale which is a 3-item scale with higher scores indicative of higher perceived risk of lung cancer; *total perceived benefits* (Cronbach α=.90), with Perceived Benefits of Lung Cancer Screening Scale which is a 6-item scale with higher scores reflective of higher perceived benefits of lung cancer screening (Cronbach α=.68); *total perceived barriers*, with Perceived Barriers to Lung Cancer Screening Scale which is a 17-item scale where higher scores reflect higher perceived barriers to lung cancer screening (Cronbach α=.86); and *total self-efficacy*, with Self-Efficacy for Lung Cancer Screening Scale and is a 9-item scale to assess individual beliefs about ability to arrange and complete an low-dose computed tomography to screen for lung cancer with higher scores reflective of higher levels of self-efficacy for lung cancer screening (Cronbach α=.83).

### Statistical Analysis

Deidentified data collected via REDCap were exported for analyses. Data completeness was assessed through descriptive analyses. Means and standard deviations or frequency distributions were examined to check for coding errors and out-of-range values.

**Figure 1:**
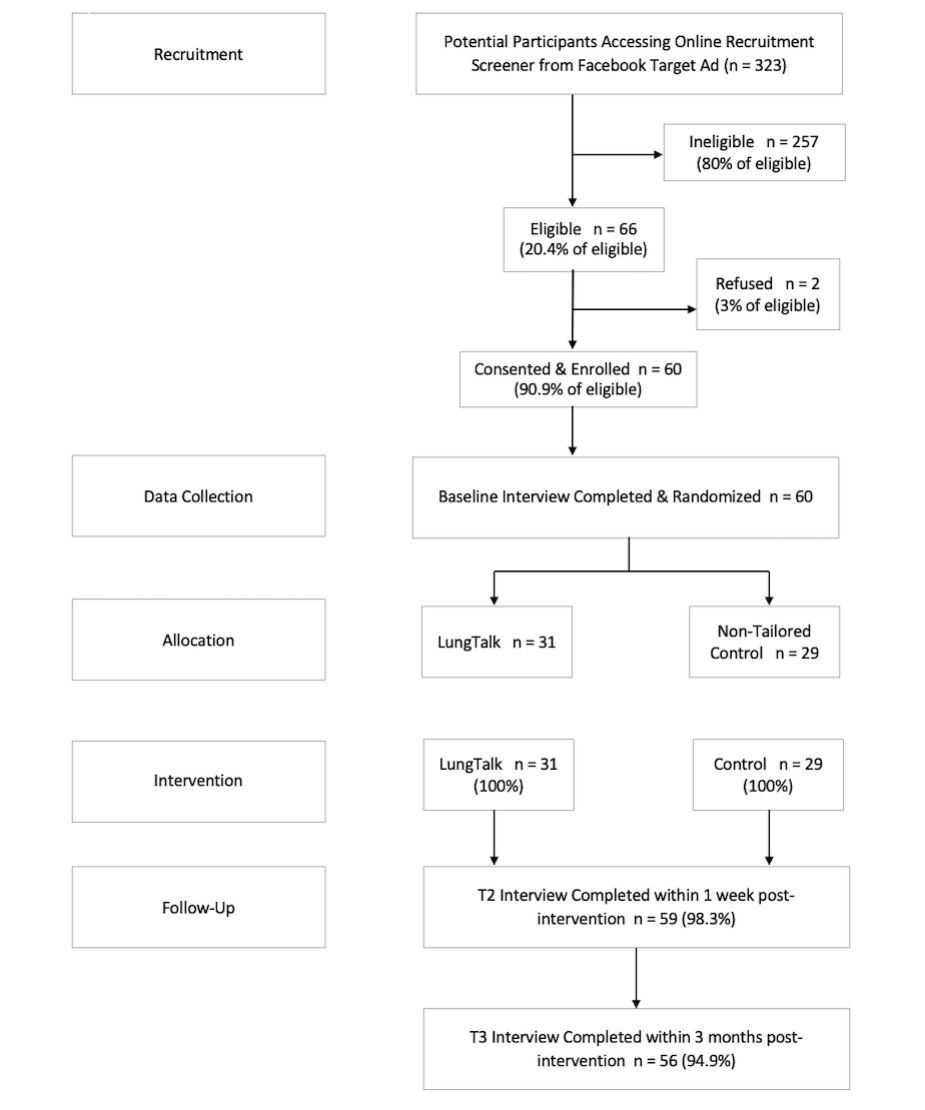
Participant recruitment flowchart.

Our first goal was to evaluate the feasibility of study procedures. Therefore, we calculated study participation rates and rates of completion and retention of participants at baseline (T1), 1-week postintervention (T2), and 3 months postintervention (T3). For each, we calculated the proportion of people who were recruited initially and retained at each data collection timepoint (see Figure 1). Patterns of missing values were examined and evaluated for randomness as described by Enders [15]. Diagnostic plots and inferential tests for tenability of assumptions were evaluated, and appropriate remedial methods were applied where required. We calculated means and standard deviations for each of the key study variables at each time point, as well as change scores calculated by value at T2 minus the value at T1, for both intervention groups. Means and standard deviations of study variables by group were also calculated.

Analysis of covariance models (ANCOVAs) were performed to determine if there were significant differences in change scores between groups, adjusted for the value at T1. Using the model result’s means and standard error, we calculated the pooled model-based standard deviation and Cohen *d* effect sizes (ie, difference between groups on the mean change scores of study variables divided by the model-based pooled SD of change scores). All analytic assumptions were verified, and analyses were performed using SAS (version 9.4; SAS Institute). Of the 60 participants recruited, 31 were randomized to receive LungTalk and 29 were randomized to the nontailored lung screening information sheet.

## Results

### Participant Sociodemographics

Participants ranged in age from 55 to 74 years (mean 62.4, SD 5.2) with fairly equal numbers of men (29/60, 48%) and women (31/60, 52%). The majority were White (48/60, 80%). Participant sociodemographic and health status characteristics are shown by intervention group in Table 1. No significant differences in sociodemographic or smoking status characteristics were observed. To assess feasibility of study procedures, Figure 1 shows the flow of participants through the study. We interviewed 100% (31/31) of group 1 (LungTalk) and 98.3% (28/29) of group 2 (nontailored lung screening information sheet) within 1 week of intervention completion.

**Table 1:** Demographics and smoking characteristics at baseline.

Characteristic		Overall (n=60)	LungTalk (n=31)	Nontailored lung screening information sheet (n=29)	*P* value
Age (years), mean (SD)		62.2 (5.2)	61.2 (4.8)	63.2 (5.5)	.13^a^
**Age (years), n (%)**					.07^b^
	55-64	38 (63)	23 (74)	15 (52)	
	65+	22 (37)	8 (26)	14 (48)	
**Sex, n (%)**					.99^b^
	Male	29 (48)	15 (48)	14 (48)	
	Female	31 (52)	16 (52)	15 (52)	
**Race, n (%)**					.86^c^
	White	48 (80)	24 (77)	24 (83)	
	Black	10 (17)	6 (19)	4 (14)	
	Other	2 (3)	1 (3)	1 (3)	
**Family history of lung cancer, n (%)**					.73^c^
	Yes	10 (17)	6 (19)	4 (14)	
	No	50 (83)	25 (81)	25 (86)	
**Smoking status, n (%)**					.78^b^
	Former	28 (46.7)	15 (48.4)	13 (44.8)	
	Current	32 (53.3)	16 (51.6)	16 (55.2)	
**Education, n (%)**					.23^c^
	<High school	1 (2)	1 (3)	0 (0)	
	High school/GED	19 (32)	10 (32)	9 (31)	
	Some college	19 (32)	12 (39)	7 (24)	
	College graduate	21 (35)	8 (26)	13 (45)	
**Income, n (%)**					
	<$25,000	25 (42)	14 (45)	11 (39)	.94^b^
	$25,000-$50,000	20 (34)	9 (29)	11 (39)	
	>$50,000	14 (24)	8 (26)	6 (21)	
**Health insurance, n (%)**					.33^c^
	Medicare	14 (24)	9 (30)	5 (18)	
	Medicaid	8 (14)	4 (13)	4 (14)	
	Private	18 (31)	11 (37)	7 (25)	
	Medicare+supplement	8 (14)	2 (7)	6 (21)	
	Other	2 (3.5)	0 (0)	2 (7)	
	Multiple	8 (14)	4 (13)	4 (14)	
Pack-years tobacco smoking, mean (SD)		48.7 (19.5)	47.6 (21.9)	49.9 (16.6)	.29^a^
Packs smoked daily, mean (SD)		1.3 (0.5)	1.4 (0.5)	1.3 (0.5)	.97^a^
Years smoked, mean (SD)		36.6 (8.3)	35.4 (9.3)	37.9 (7.0)	.10^a^
Years since quitting, mean (SD)		6.3 (5.0)	6.7 (5.7)	5.8 (4.4)	.77^a^

^a^Wilcoxon rank-sum test.

^b^Chi-square test.

^c^Fisher exact test.

### Changes in Knowledge

Mean scores, standard deviations, and change scores for knowledge and beliefs are shown in Table 2, along with the effect sizes and tests of the within-group change. Knowledge scores increased significantly for both groups from baseline to postintervention, although the improvement was greater for the group receiving LungTalk (2.33 vs 1.14 change in means which represents a 1.5 SD vs 1 SD change). Perceived self-efficacy for lung cancer screening also increased significantly for both groups, with a 0.5 SD change for each group. A small reduction in perceived risk of getting lung cancer was observed in the LungTalk group (SRM –0.13 or about one-tenth of an SD decrease) while an increase in perceived risk was seen in the nontailored lung screening information sheet group (SRM 0.30 or about one-third of an SD increase). Perceived benefits improved significantly for the LungTalk group (SRM 0.41, almost one-half SD increase), whereas the minor decrease in benefits was not significant for the nontailored lung screening information sheet group (SRM –0.14). A nonsignificant reduction in perceived barriers was observed in both groups, although the reduction was greater for the enhanced control group (SRM –0.33 vs –0.15).

While Table 2 provides insight into the within-group effects from each group separately, estimates of efficacy of LungTalk on knowledge and health beliefs are presented in Table 3. In Table 3, the between-group effect sizes and *P* values are shown for comparing groups on changes from baseline to 1-week postintervention on total scale scores, controlling for T1 value of the score (ie, controlling for initial score level at baseline). Compared with the nontailored lung screening information sheet group, the LungTalk group had a greater increase in total knowledge scale scores (8 items with *P*<.01). The large effect size of 0.85 indicated that the increase in knowledge for those who received LungTalk was more than three-quarters of an SD greater than the increase in knowledge for those who received the nontailored lung screening information sheet. While the change in perceived benefits did not reach significance, change was occurring in the theoretically expected direction. LungTalk increased participants’ perceptions of the benefits of lung cancer screening while benefits scores decreased slightly for the lung screening information sheet group (1.07 vs –0.18, *P=*.06).

Because knowledge was significantly higher for those who received LungTalk, we examined group differences on individual knowledge items to see where specific improvements were made (Table 4). Interestingly, the percentage of participants who correctly answered that a low-dose computed tomography of the chest is the test that is currently recommended for lung cancer screening increased significantly from baseline in both groups (LungTalk: 57% increase, lung screening information sheet: 52% increase; *P<*.001). Likewise, the percentages of participants in both groups who correctly knew that lung cancer screening is only recommended for current and former smokers increased significantly from baseline (LungTalk: 38%, *P=*.004; lung screening information sheet: 24%; *P=*.008). Compared to the lung screening information sheet group, significantly greater improvements in knowledge were seen for those receiving LungTalk on 3 knowledge items: (1) knowing that a person should talk with their health care provider about lung cancer screening before being screened (*P=*.10 vs *P=*.01, respectively); (2) knowing that lung cancer screening, if results are normal, should be done annually (*P=*.71 vs *P=*.007, respectively); and (3) knowing that 55 is the age that people should start screening for lung cancer (*P=*.48 vs *P*<.001, respectively).

**Table 2:** Scores at baseline (T1) and 1-week postintervention (T2) and within-group tests.

Variable		Lung Talk, mean (SD)	Nontailored lung screening information sheet, mean (SD)
**Total knowledge score**			
	T1	3.90 (1.47)	3.66 (1.47)
	T2	6.27 (1.26)	4.79 (1.32)
	Change	2.33 (1.54)	1.14 (1.16)
	SRM^a^	1.51	0.98
	*P* value^b^	<.01	<.01
**Total perceived risk**			
	T1	13.74 (2.66)	13.69 (1.95)
	T2	13.43 (2.40)	14.28 (2.36)
	Change	–0.27 (2.13)	0.59 (1.96)
	SRM	–0.13	0.30
	*P* value^b^	.50	.12
**Total perceived benefits**			
	T1	17.55 (1.88)	18.34 (2.70)
	T2	18.70 (3.10)	18.07 (2.89)
	Change	1.17 (2.85)	–0.28 (2.00)
	SRM	0.41	–0.14
	*P* value^b^	.03	.46
**Total perceived barriers**			
	T1	34.10 (7.15)	33.03 (6.48)
	T2	33.20 (7.43)	30.90 (7.24)
	Change	–0.90 (5.86)	–2.14 (6.49)
	SRM	–0.15	–0.33
	*P* value^b^	.41	.09
**Total self-efficacy**			
	T1	27.45 (5.08)	28.38 (4.87)
	T2	28.97 (4.55)	30.38 (4.16)
	Change	1.53 (3.30)	2.00 (3.56)
	SRM	0.46	0.56
	*P* value^b^	.02	.01

^a^SRM: standardized response mean = mean change / SD of change.

^b^2-sided paired test.

**Table 3:** Effect sizes.

Variable	Scale range	Adjusted mean (SE) change		Comparison			
		LungTalk	Nontailored lung screening information sheet	*F* test (*df1*, *df2*)	*P* value	Mean difference (95% CI)	Cohen *d* effect size^a^
Total knowledge	0-6	2.41 (0.20)	1.06 (0.21)	21.5 (1, 56)	<.01	1.35 (0.77, 1.93)	0.8482
Total perceived risk	3-12	–0.26 (0.34)	0.58 (0.35)	3.03 (1, 56)	.09	–0.85 (–1.83, 0.13)	–0.3179
Total perceived benefits	6-24	1.07 (0.45)	–0.18 (0.45)	3.79 (1, 56)	.06	1.25 (–0.04, 2.54)	0.3555
Total perceived barriers	17-68	–0.72 (1.06)	–2.32 (1.07)	1.12 (1, 56)	.29	1.60 (–1.43, 4.62)	0.1931
Total self-efficacy	9-36	1.37 (0.54)	2.17 (0.55)	1.08 (1, 56)	.30	–0.80 (–2.35, 0.75)	–0.1896

^a^Positive effect size indicates greater increase from T1 to T2 for LungTalk than for InfoSheet. Negative effect size indicates greater increase from T1 to T2 for InfoSheet than for LungTalk.

**Table 4:** Individual knowledge items by within-group change.

Knowledge item	LungTalk			Nontailored lung screening information sheet		
	Baseline, n (%) correct	T2, n (%) correct	*P* value^a^	Baseline, n (%) correct	T2, n (%) correct	*P* value^a^
Who is more likely to get lung cancer? (a person who has smoked cigarettes for a long time)	27 (87.1)	28 (93.3)	.41	26 (89.7)	27 (93.1)	.56
What is the most common symptom of lung cancer? (chronic cough)	25 (80.7)	25 (83.3)	.56	24 (82.8)	27 (93.1)	.18
Which test is currently recommended for lung cancer screening? (low-dose CT^b^ scan)	8 (25.8)	25 (83.3)	<.001	9 (31.0)	24 (82.8)	.001
Compared to a chest x-ray, how much radiation does a lung scan expose you to? (about the same as a chest x-ray)	6 (19.4)	11 (36.7)	.06	6 (20.7)	8 (27.6)	.41
What should a person do before being screened for lung cancer? (talk with their health care provider about low-dose CT screening)	21 (67.7)	29 (96.7)	.01	20 (69.0)	24 (82.8)	.10
If you choose to have a lung scan to screen for lung cancer and everything is normal, when will you need to have your next one? (in 1 year)	17 (54.8)	25 (83.3)	.007	8 (27.6)	7 (24.1)	.71
Who is currently recommended to have a lung scan to screen for lung cancer? (only current and former smokers)	10 (32.3)	21 (70.0)	.004	6 (20.7)	13 (44.8)	.008
At what age is it recommended that people start to screen for lung cancer? (55)	7 (22.6)	24 (80.0)	<.001	7 (24.1)	9 (31.0)	.48

^a^2-sided McNemar test of paired proportions.

^b^CT: computed tomography.

### Changes in Participants’ Perceptions

As shown in Table 5, satisfaction with the LungTalk intervention was significantly higher than with the nontailored lung sreening information sheet. Individuals in both groups felt “prepared” or “very prepared” to have a discussion with their clinician about lung screening, with no significant differences between the 2 intervention groups on preparedness (*P*=.52).

**Table 5:** User satisfaction, clinician recommendation, shared decision-making discussion by group.

Variable		Lung Talk (n=31)		Nontailored lung screening information sheet (n=29)		*P* value^a^	
**Satisfaction (T2)**						.002	
	Not at all satisfied		0 (0)		1 (3)		
	Somewhat satisfied		1 (3)		4 (14)		
	Satisfied		7 (23)		16 (55)		
	Very satisfied		22 (73)		8 (28)		
**Preparedness (T2)**						.52	
	Somewhat prepared		4 (13)		6 (21)		
	Prepared		10 (33)		6 (21)		
	Very prepared		16 (53)		17 (59)		
**Clinician recommendation (T3)**						.33	
	Yes		8 (28)		4 (15)		
	No		22 (72)		25 (85)		
**Shared decision-making discussion about lung cancer screening (T3)**						.23	
	Yes		10 (34)		5 (19)		
	No		20 (66)		24 (81)		

^a^2-sided Fisher exact test.

P-value is from two-sided Fisher’s exact Test.

### Changes in Self-Reported Patient-Clinician Discussions

At 6 months, though the number of participants who reported that they had a discussion with their clinician in the LungTalk group (10/31, 34.5%) was double that in the nontailored lung screening information sheet group (5/29, 18.5%), this difference was not significant (*P=*.23). Similar results were observed at 6 months for receipt of a clinician recommendation for lung screening; 27.6% (8/31) in the LungTalk group reported receiving a recommendation from their clinician compared to 14.8% (4/29) in the nontailored lung screening information sheet group. This difference was also not significant (*P=*.33).

## Discussion

### General

It is still relatively early on in the development of decision support tools for lung cancer screening. Most have focused on calculating personal risk for the development of lung cancer and subsequent recommendations to screen are based upon calculated risk status [16-20]. These tools range in level of complexity and delivery including pamphlets, brochures, videos, educational scripts, and computer programs [16-20]. These tools can also be deployed in multiple formats such as by mail, telephone, in person, and via the internet. Dharod and colleagues [14] examined the feasibility of a digital health outreach strategy via a patient portal directing individuals to an interactive website which then accessed screening eligibility.

Similar to other patient decision aids [16-20], the mPATH (mobile Patient Technology for Health) Lung Interactive website is atheoretical and calculates risk for lung cancer but does not tailor beyond personalized risk [14]. LungTalk focuses on empowering individual patients with knowledge so that they are an informed partner as they discuss with their clinician and make a decision about lung cancer screening. In addition, LungTalk goes beyond much of what other decision tools focus on by addressing lung health broadly, in the context of performing a low-dose computed tomography of the chest that has the potential to uncover other conditions of concern beyond lung cancer, to more comprehensively educate the individual. LungTalk extends the work of nontailored decision aids in the field, by introducing tailoring algorithms and message framing based upon smoking status in order to determine what components of the intervention are driving behavior change when an individual is informed and makes the decision to screen, or not, for lung cancer. LungTalk is innovative in its tailored messaging approach based on smoking status and tailored printout that helps patients initiate a discussion with their clinician about their lung health and the option of screening.

As an intervention, individuals using LungTalk felt equally prepared to engage in a discussion with their clinician about lung cancer screening as they did with the nontailored lung screening information sheet. From the patient perspective, being prepared to engage in a shared discussion about lung cancer screening is essential to successfully involving the patient in the dyadic communication clinical context of this patient-clinician equitable engagement; however, participants were significantly more satisfied with LungTalk (*P*=.002). As interventions are developed, it is essential for developers to take into consideration user satisfaction in efforts to increase the likelihood of both initial and sustained engagement with the intervention.

Consistent with other types of cancer screening, knowledge and health beliefs have been shown to be associated with lung cancer screening behavior [11]. In addition, there is strong support in other types of cancer screening that tailored interventions are much more effective in promoting cancer screening behavior because messaging is more personally relevant to the individual making the decision to screen. LungTalk included messaging to increase knowledge and results support its effectiveness in doing so over the nontailored intervention (8 item with *P*<.01). In particular, knowing that normal or negative lung cancer screening results still require adherence to annual screening while eligible is important and LungTalk increased this knowledge level compared to the change from the nontailored lung screening information sheet (*P=*.007). Since the majority of lung cancers identified on lung cancer screening exams were on subsequent exams as opposed to initial exams, this is essential knowledge for screening-eligible individuals.

With regard to health beliefs, even though LungTalk improved perceived benefits and self-efficacy in expected directions, changes were not significantly different compared those of the lung screening information sheet. LungTalk fell short in reducing perceived barriers to lung cancer screening, an important variable that often predicts cancer screening behaviors. The impact of these interventions on perceived risk of lung cancer was not observed and highlights a critical gap in the tailored messaging component of the intervention. Moving forward, it is important that tailored messages be further refined to target specific barriers to lung cancer screening as well as perceived risk as it relates to lung cancer for the target patient population in order to improve the efficacy of LungTalk as both a health communication and decision support tool.

### Strengths and Limitations

As a pilot study, we had adequate power for estimating effect sizes and detecting large effect sizes. For example, 26 participants in each group were required for 80% power to detect a large Cohen *d* effect size of 0.80 SD difference between means, and we slightly exceeded 26 per group. A larger sample (eg, 64 per group) would be needed to detect a medium effect size of 0.50 with 80% power.

As with all studies, this study is not without limitations. Our recruitment methods may have influenced who participated in this study. Targeted Facebook advertisement allowed us to purposively sample people aged 55 years and older who indicated smoking as an interest on their Facebook page. People who use Facebook and who indicate smoking as an interest may constitute unique sample. Our sample demographics, however, indicate that we successfully recruited a racially diverse, national sample with equal numbers of men and women. Randomization also was effective; no differences between groups were observed at baseline. Our nonsignificant group differences in change scores on health belief variables were likely due to inadequate integration of content and tailored messages in LungTalk that would impact health belief constructs. Future versions of LungTalk need to address these constructs specifically in content and tailored messages if we are to change health beliefs in the directions that promote patient-clinician discussions about lung cancer screening and shared decisions.

### Conclusion

Preliminary results indicate LungTalk is a helpful communication tool for individuals who are considering the option of lung cancer screening. Specifically, LungTalk can help enhance the shared decision-making process by priming individuals with essential baseline knowledge to support an informed discussion with a health care clinician about potential risks and benefits related to lung cancer screening.

## Appendix

Multimedia Appendix 1 Screenshots - LungTalk.

## Abbreviations

SRM: standardized response mean
USPSTF: US Preventive Services Task Force
