# Supplementary material for: Computer-Tailored Decision Support Tool for Lung Cancer Screening: Community-Based Pilot Randomized Controlled Trial
Source: J Med Internet Res. 2020 Nov 3;22(11):e17050. doi: 10.2196/17050 (PMC7671845; doi:10.2196/17050)
Supplement: Multimedia Appendix 1 [file jmir_v22i11e17050_app1.pdf]

comet.soic.iupui.edu

LungTalk

Start

Welcome to Lung Talk!

comet.soic.iupui.edu

LungTalk

About You...

Welcome

Introduction

Your Lungs

About You...

Please tell us if you currently smoke or have quit smoking?

I currently smoke | I quit smoking

Cigarette Smoke: What It Does

Welcome
Lung Diseases
Cigarette Smoke
Lung Cancer
No Symptoms
Symptoms
Talk About It
Summary

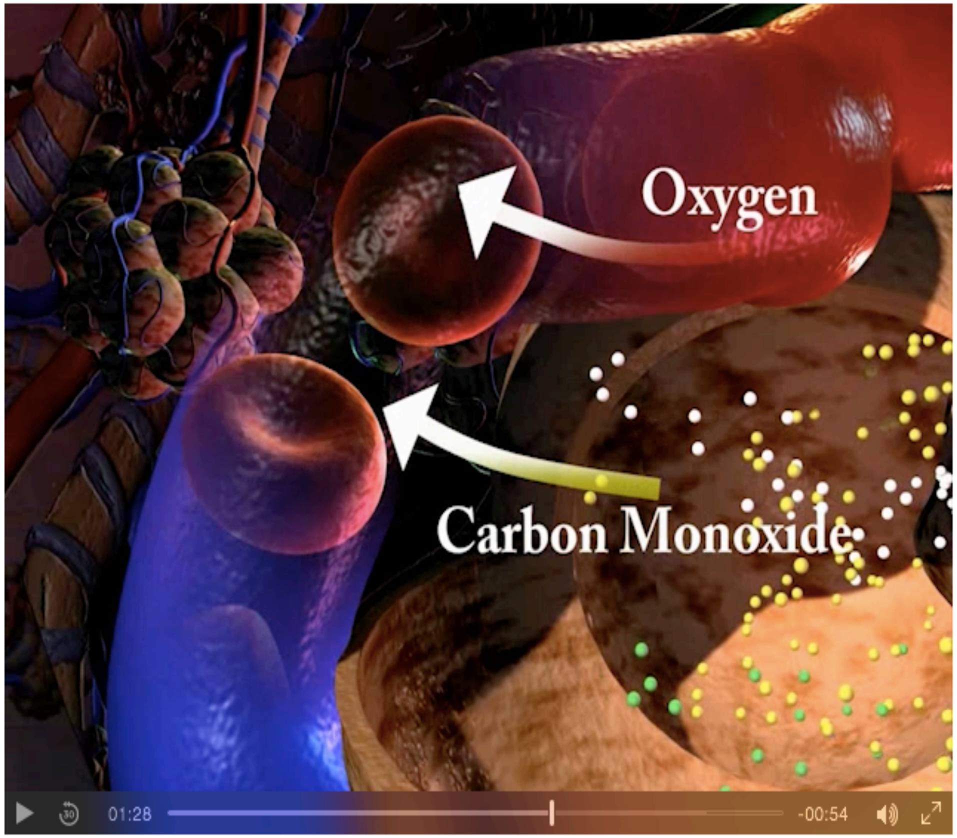

Emphysema

LungTalk

Cigarette Smoke: What It Does

- Welcome
- Lung Diseases
  - Cigarette Smoke
  - Lung Cancer
  - No Symptoms
  - Symptoms
- Talk About It
- Summary

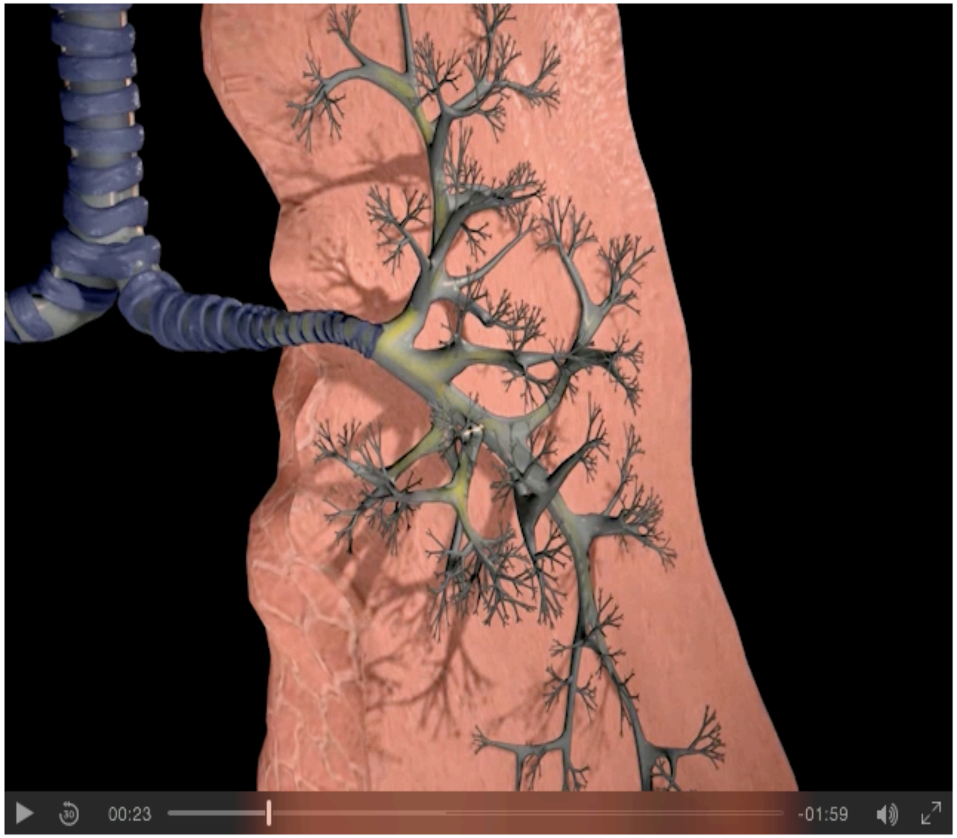

Emphysema

LungTalk

Lung Scan

Welcome
Lung Diseases
Talk About It
- Lung Screening
- Lung Scan
- Benefits
- Things to Consider
- Possible Results

Summary

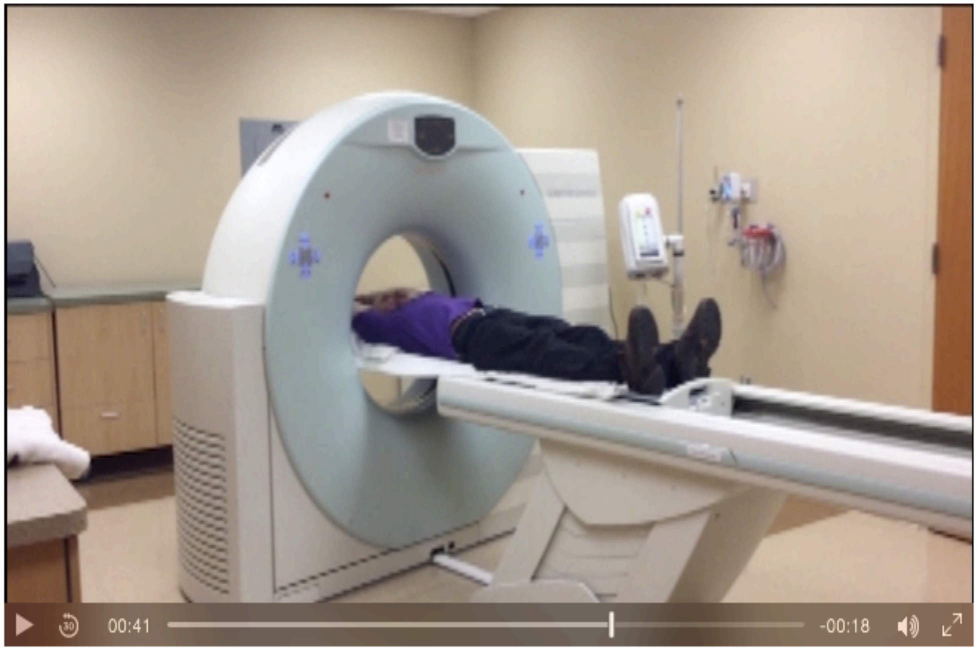

What I Would Like To Talk About

Welcome
Lung Diseases
Talk About It
Summary
To Talk About
LungTalk Printout
More Information

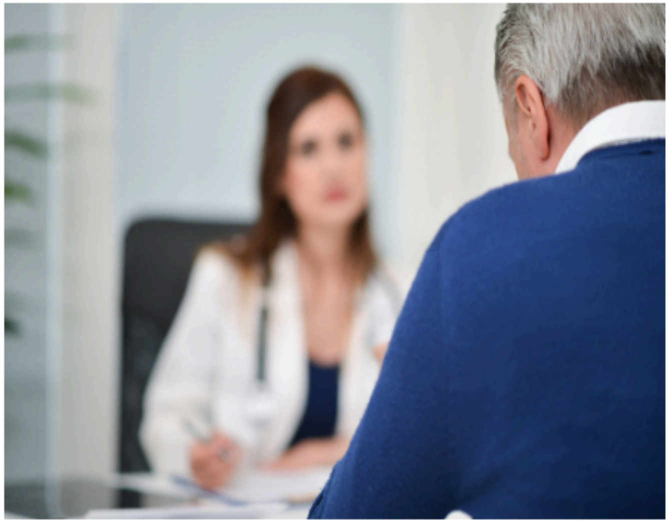

I would like to talk about (choose up to 3):

- ☐ My personal risk of lung cancer.
- ☐ More information about the lung scan.
- ☐ Benefits of lung screening.
- ☐ False positives, over-diagnosis, and radiation exposure.
- ☐ What my doctor recommends for me.
- ☐ How long I would have to do yearly lung scans.
- ☐ Ways to stop smoking.
- ☐ Other

Please click the right arrow when you are ready.
